# fNIRS Studies on Hemispheric Asymmetry in Atypical Neural Function in Developmental Disorders

**DOI:** 10.3389/fnhum.2017.00137

**Published:** 2017-04-12

**Authors:** Hirokazu Doi, Kazuyuki Shinohara

**Affiliations:** Department of Neurobiology and Behavior, Graduate School of Biomedical Sciences, Nagasaki UniversityNagasaki, Japan

**Keywords:** fNIRS, hemispheric asymmetry, ADHD, ASD, prefrontal cortex, latealization

## Abstract

Functional lateralization is highly replicable trait of human neural system. Many previous studies have indicated the possibility that people with attention-deficits/hyperactivity-disorder (ADHD) and autism spectrum disorder (ASD) show hemispheric asymmetry in atypical neural function. However, despite the abundance of relevant studies, there is still ongoing controversy over this issue. In the present mini-review, we provide an overview of the hemispheric asymmetry in atypical neural function observed in fNIRS studies on people with these conditions. Atypical neural function is defined as group-difference in the task-related concentration change of oxygenated hemoglobin. The existing fNIRS studies give support to the right-lateralized atypicalty in children with ADHD. At the same time, we did not find clear leftward-lateralization in atypical activation in people with ASD. On the basis of these, we discuss the current states and limitation of the existing studies.

## Lateralization in atypical neural function in developmental disorders

Functional near-infrared spectroscopy (fNIRS) was introduced into the scientific community as a neuroimaging tool ~20 years ago (Hoshi and Tamura, 1993; Kato et al., 1993). Despite having relatively poor spatial and temporal resolution compared to fMRI and EEG/MEG respectively, fNIRS is associated with certain advantages over other non-invasive techniques for measuring neural function. For instance, fNIRS poses a low physical and psychological burden on participants. Additionally, fNIRS is less vulnerable to artifacts generated by bodily motion. These features are particularly advantageous for measuring neural function in individuals with pathological conditions (Doi et al., 2013; Koike et al., 2013; Adorni et al., 2016).

Besides them, fNIRS has some unique characteristics compared to the other non-invasive measurements of neural function. First, in contrast to EEG, which measures the electrical activity (primary signal) pooled across wide neural regions, fNIRS measures hemodynamic response (secondary signal) with relatively high spatial resolusion. Second, the concentrations of oxygenated-/deoxygenated hemoglobin (oxyHb/deoxyHb) measured by fNIRS reflect aspects of hemodynamic response that are different from the indicators used in other neuroimaging techniques (Minagawa-Kawai et al., 2009a). Relative increase of oxyHb concomitant with slight decrease of deoxyHb is supposed to reflect the influx of oxyHb to the blood vessels adjascent to activated cortical region to meet the demands of energy consumption by neurons in the region (Minagawa-Kawai et al., 2009a; Doi et al., 2013). In contrast to this, the BOLD signal measured in fMRI technique is considered to mainly reflect the decrease of deoxyHb (Song et al., 2006), although the physiological basis of BOLD signal remains elusive at this point. Therefore, incorporating findings from fNIRS studies might lead to a more comprehensive understanding of typical and atypical patterns of neural function.

Functional lateralization has been repeatedly documented in the human neural system; a number of studies have generally shown leftward-lateralization of linguistic function (Crow, 2000) and right-lateralization of attentional function and visuo-spatial cognition (Toga and Thompson, 2003; Hervé et al., 2013). There is a long history of studies investigating lateralization in atypical neural function in developmental disorders (McCann, 1982; see, Klimkeit and Bradshaw, 2006, for a brief review). However, despite the abundance of relevant studies, there is still controversy over whether people with developmental disorders exhibit lateralization in atypical neural function.

Since its introduction, the number of fNIRS studies focused on people with developmental disorders has been steadily increasing (for a review, Ehlis et al., 2014). Because the majority of these studies have used bilaterally-placed multichannel emitter-detector probe sets, the resulting datasets offer an invaluable opportunity to examine lateralization in atypical neural function in individuals with developmental disorders.

## Aim

Here we provide a qualitative overview of the existing fNIRS studies of individuals with developmental disorders, with a specific focus on lateralization in atypical neural function. Although several reviews of fNIRS research have been published (Doi et al., 2013; Koike et al., 2013; Ehlis et al., 2014; Balconi et al., 2015; Adorni et al., 2016), to the best of our knowledge, this is the first to focus on this aspect. The conditions discussed here include attention-deficit/hyperactivity disorder (ADHD) and autism spectrum disorder (ASD). Previous studies have indicated the possibility that individuals with these conditions (McCann, 1982; Klimkeit and Bradshaw, 2006) show lateralization in atypical neural function, but these findings are not often consolidated into theoretical overviews. Therefore, our primary goal here is to establish a scaffolding for the organization and consolidation of findings obtained using fNIRS regarding the lateralization in atypical neural function in people with ADHD and ASD.

As per convention, we treat task-related increases in oxyHb as the primary indicator of neural function (Minagawa-Kawai et al., 2009a; Doi et al., 2013). The atypicality of neural function observed so far comes mainly in three forms. First, some studies have quantitatively compared oxyHb changes between patient and control groups. As a result, many studies found statistically significant between-group differences in the level of task-related oxyHb change in either one or both hemispheres. In the second type of atypicality, hemispheric asymmetry is observed in either the patient or control group, but not in both. More specifically, in some such cases the patient group does not show the lateralized oxyHb changes observed in the control group (lack of lateralization), while in others patients show lateralization not normally observed in matched-controls. Third, several studies have revealed a lack of significant task-related changes in oxyHb from the preceding baseline period in the patient group in either one of the hemispheres when matched-controls showed significant task-related changes.

Of the three types of atypicality described above, we focus mainly on the first, as only this type is ascertained by the direct comparison of patient and matched-control groups. For descriptive brevity, we refer to such reduced/enhanced levels of task-related oxyHb increase in patients compared with matched controls as “hypo-/hyper-activation.” In the following, the term “lateralization in atypical neural function/activation” refers mainly to hypo-/hyper-activation being observed only in one hemisphere.

## Lateralization in atypical neural function in ADHD

ADHD is a developmental disorder with inattention, impulsivity, and hyperactivity as core symptoms. Children with ADHD often have poor social skills and learning disabilities. Approximately 10% of school-aged children and 5% of adults are estimated to suffer from ADHD (Pietrzak et al., 2006; Safren et al., 2010; Thomas et al., 2015).

It has long been postulated that the symptoms of ADHD are associated with right-hemisphere abnormalities (Stefanatos and Wasserstein, 2001). This is largely because functions such as attentional control, visuo-spatial processing, and socio-emotional processing, for which ADHD children show relatively poor performance, are generally right-lateralized in typically developing people (Toga and Thompson, 2003; Hervé et al., 2013). This notion has gained support from studies utilizing behavioral experiments and neuroimaging techniques (for review, Stefanatos and Wasserstein, 2001; Valera et al., 2007). However, several recent studies have shown a more nuanced pattern of atypical lateralization (Silk et al., 2016) or have shown atypical interhemispheric integration (Hale et al., 2009) in ADHD.

**Figure 1:**
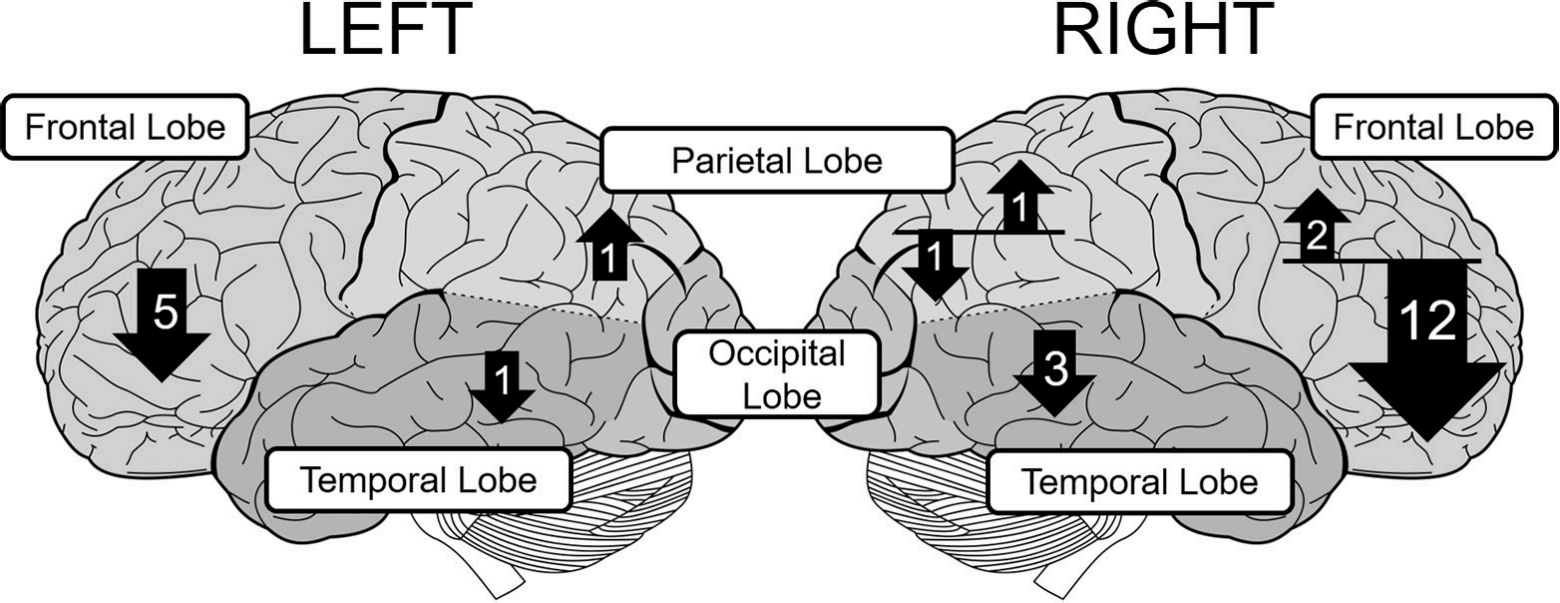
**The distribution of group-differences in each lobe in children with ADHD**. The upward and downward arrows represent hyper- and hypoactivation, respectively. The number in each arrow represents the number of papers that found statistically significant group difference. The size of each arrow is not strictly proportional to the number of papers.

The number of fNIRS studies of individuals with ADHD is relatively small, but these generally support right-lateralized atypicality in wide cortical regions in ADHD. To further verify this observation, we surveyed relevant peer-reviewed studies using the Scopus database. We mainly included studies that compared task-related oxyHb changes in bilaterally-placed channels, between people with ADHD and matched controls. Conference proceedings and review papers were excluded. This resulted in a total of 24 eligible studies. The details of these studies are summarized in Table 1. The distribution of observed group-differences are described in Figure 1.

**Table 1:** **The details of the main fNIRS studies on people with ADHD explained in the present mini-review: only the results of group comparison with matched-controls are shown**.

		**Age**	**Model of NIRS machine**	**Number of channels**	**Task requirements**	**Measured regions**	**Dependentvariables**	**Main analysis offNIRS data**
Children	Weber et al., 2005	11 boys with ADHD (*M* = 10.4 ± 1.2) 9 TDC (9 boys; *M* = 11.3 ± 1.3)	NIRO-300	2	Trail-making task	Frontal lobe	OxyHb DeoxyHb Cytochrome oxidase aa3 Tissue oxygenation index Cerebral blood volume	Test of task-related change of dependent variables from baseline Group difference of dependent variables
	Jourdan Moser et al., 2009	12 boys with ADHD (*M* = 10.1 ± 1.9) 12 TDC (12 boys; *M* = 10.6 ± 1.6)	NIRO-300	4	Stroop task	Frontal lobe	OxyHb DeoxyHb Behavioral performance	Channel-wise analysis of group difference and task-related change from baseline
	Schecklmann et al., 2010	2 girls and 17 boys with ADHD (*M* = 11.6 ± 14.4) 19 TDC (4 girls and 15 boys; *M* = 11.6 ± 13.7)	ETG-4000	52	Spatial working memory task	Frontal lobe	OxyHb Behavioral performance	ANOVA on the mean oxyHb values in six ROIs (left/right VLPFC, DLPFC, SFS) with the factors of Group × Hemisphere × Condition
	Negoro et al., 2010	2 girls and 18 boys with ADHD (*M* = 9.55 ± 1.93) 20 TDC (17 boys and 3 girls; *M* = 9.35 ± 2.13)	ETG-100	24	Stroop color word task	Frontal lobe	OxyHb	Channel-wise analysis of group difference
	Schecklmann et al., 2011a	7 girls and 20 boys with ADHD(*M* = 12.7 ± 1.4) 21 TDC (14 girls and 8 males; *M* = 12.4 ± 1.6)	ETG-4000	24	Olfactory stimulation	Frontal lobe, Temporal lobe	OxyHb DeoxyHb Olfactory test score	Test of task-related change from baseline and group comparisons in each of the four ROIs (inferior frontal, temporal)
	Xiao et al., 2012	16 boys with ADHD (*M* = 9.75 ± 1.18) 16 TDC (16 boys; *M* = 9.69 ± 1.74)	JH-NIRS-BR-05	16	Go/NoGo task Stroop task	Frontal lobe	OxyHb Behavioral performance	Group comparison of mean oxyHb by *t*-tests
	Inoue et al., 2012	6 girls and 14 boys with ADHD (*M* = 9.6) 20 TDC (6 girls and 14 boys; *M* = 9.7)	Cognoscope	16	Go/NoGo task	Frontal lobe	OxyHb DeoxyHb Behavioral performance	ANOVA on mean oxyHb values in four ROIs (left/right medial/lateral) with the factors of Group × ROI × Condition
	Tsujimoto et al., 2013	16 boys with ADHD (*M* = 10.9 ± 2.0) 10 TDC (10 boys; *M* = 10.1 ± 1.8)	OEG-16	16	Spatial working memory task With/without attentional distractor	Frontal lobe	OxyHb Behavioral performance	ANOVA on mean oxyHb values in three ROIs (left/middle/right PFC) with the factors of ROI × Group
	Yasumura et al., 2014	2 girls and 8 boys with ADHD (*M* = 11.8 ± 2.23) 15 TDC (6 boys and 9 girls; *M* = 9.56 ± 1.51)	OEG-16	16	Stroop task Reverse stroop task	Frontal lobe	OxyHb SNAP questionnaire Behavioral performance	ANOVA on mean oxyHb in each hemisphere with the factors of Hemisphere × Group
	Ichikawa et al., 2014	13 boys with ADHD (*M* = 10.0 ± 1.3) 13 TDC (13 boys; *M* = 9.7 ± 1.3)	ETG-4000	24	Passive viewing of emotional faces	Temporal lobe	OxyHb DeoxyHb Total Hb Timing of peak activation	ANOVA with the factors of Group × Hemisphere × Condition Test of task-related increase from baseline Variance test of peak latency of oxyHb
	Nagashima et al., 2014a	19 boys and 3 girls with ADHD (*M* = 9.5 ± 2.0) 22 TDC (15 boys and 7 girls; *M* = 9.8 ± 2.0)	ETG-4000	22	Oddball task	Frontal lobe, Parietal lobe, Temporal lobe	OxyHb DeoxyHb Behavioral performance	Channel-wise analysis of group difference between control, post-/pre-medicated ADHD Channel-wise analysis of task-related oxyHb change from baseline
	Nagashima et al., 2014b	3 girls and 12 boys with ADHD (*M* = 9.9 ± 2.1) 15 TDC (12 boys and 3 girls; *M* = 10.1 ± 1.7)	ETG-4000	22	Oddball task	Frontal lobe, Parietal lobe, Temporal lobe	OxyHb DeoxyHb Behavioral performance	Channel-wise analysis of group difference between control, post-/pre-medicated ADHD Channel-wise analysis of task-related oxyHb change from baseline
	Nagashima et al., 2014c	2 girls and 14 boys (*M* = 8.8 ± 2.2) 16 TDC (14 boys and 2 girls; *M* = 8.9 ± 2.2)	ETG-4000	22	Go/NoGo task	Frontal lobe, Parietal lobe, Temporal lobe	OxyHb DeoxyHb Behavioral performance	Channel-wise analysis of group difference between control, post-/pre-medicated ADHD Channel-wise analysis of task-related oxyHb change from baseline
	Monden et al., 2015	5 girls and 25 boys with ADHD (*M* = 9.1 ± 2.6) 30 TDC (10 girls and 20 boys; *M* = 9.7 ± 2.3)	ETG-4000	22	Go/NoGo task	Frontal lobe, Parietal lobe, Temporal lobe	OxyHb DeoxyHb Behavioral performance	Channel-wise analysis of group difference between control, post-/pre-medicated ADHD Channel-wise analysis of task-related oxyHb change from baseline ROC analysis
	Köchel et al., 2015	14 boys with ADHD (*M* = 10.4 ± 1.5) 14 TDC (14 boys; *M* = 10.2 ± 0.9)	ETG-4000	24	Emotional prosody recognition task	Parietal lobe, Temporal lobe	OxyHb DeoxyHb Behavioral performance	Group comparison of mean oxyHb values of four ROIs (left/right Parietal/Temporal region)
	Yasumura et al., 2015	7 girls and 15 boys with ADHD (*M* = 10.3 ± 2.0) 37 TDC (19 boys and 18 girls; *M* = 10.8 ± 1.6)	OEG-16	16	Dimensional card sorting task	Frontal lobe	OxyHb PARS SNAP Behavioral performance	Channel-wise analysis of group difference
	Ishii-Takahashi et al., 2015^*^	Drug naïve children with ADHD (4 girls and 18 boys; *M* = 8.6 ± 1.4) 20 TDA (14 boys and 6 girls; *M* = 8.1 ± 1.6)	ETG-4000	52	SST	Frontal lobe	OxyHb CGI-S ADHD-RS-IV CBCL Behavioral performance	ANOVA with the factors of Group × Hemisphere × Session (baseline, 4-to-8 week open trial)
	Araki et al., 2015	6 boys and 6 girls with ADHD (*M* = 9.8 ± 2.3) 14 TDC (5 boys and 9 girls; *M* = 9.7 ± 2.8)	ETG-100	24	Continuous performance test	Frontal lobe	OxyHb AHDH RS-IV-J score Behavioral performance	In the analysis of pre-/post-medication, ANOVA with the factors of channel and time-segment within each group
Adult	Ehlis et al., 2008	9 males and 4 females with ADHD (*M* = 29.8 ± 8.0) 13 TDA (8 males and 5 females; *M* = 26.8 ± 3.6)	ETG-100	24	Letter n-Back task	Frontal Lobe	OxyHb DeoxyHb Behavioral Performance	Channel-wise comparison of group difference
	Schecklmann et al., 2009	6 females and 8 males with ADHD (*M* = 40.4 ± 10.7) 14 TDA (5 females and 9 males; *M* = 40.6 ± 8.9)	ETG-4000	22	Phonological and Semantic VFT	Frontal Lobe, Parietal Lobe, Temporal Lobe	OxyHb Behavioral Performance	Group-difference for the average of active channels which showed the expected pattern of activation in both control and ADHD groups
	Schecklmann et al., 2011b	15 females and 14 males with ADHD (*M* = 28.2 ± 4.5) 29 TDA (15 females and 14 males; *M* = 27.8 ± 4.1)	ETG-4000	22	Olfactory stimulation by odors with three levels of concentration	Frontal Lobe, Temporal Lobe	OxyHb Sniffing Stick test WURS-k I7 Impulsivity scale Behavioral performance	Test of oxyHb change from zero and group difference in each of the 5 ROIs (Temporal region, Inerior Frontal region, Somatosensory region, Broca' area)
	Schecklmann et al., 2012	21 females and 24 males with ADHD (*M* = 36.4 ± 9.9) 41 TDA (21 females and 20 males; *M* = 36.1 ± 10.1)	ETG-4000	52	Working memory task SST	Frontal Lobe	OxyHb DeoxyHb I7 impulsivity WURS-k Behavioral performance	ANOVA on mean oxyHb values in ROIs defined in a data-driven manner with the factors of group × Task
	Ishii-Takahashi et al., 2014	8 females and 11 males with ADHD (*M* = 30.6 ± 7.4) 21 TDA (13 males and 8 females; *M* = 28.8 ± 5.5)	ETG-4000	52	SST VFT	Frontal Lobe, Temporal Lobe	OxyHb Behavioral Performance	Channel-wise group comparison of oxyHb Classification of groups by Linear discriminant analysis using oxyHb
		**Typical activation pattern**	**Patients compared to controls**	**Hemisphere**	**Regions with group difference**	**Other findings**		
Children	Weber et al., 2005	Significant bilateral increase of oxyHb during extended-attention	n.s	n.s	n.s	DeoxyHb increase in the left hemisphere was larger in the control group than in the AHDH group		
	Jourdan Moser et al., 2009	Significant oxyHb increase during stimulation	n.s.	n.s.	n.s.	The onset of hemodynamic response was generally delayed in children with ADHD. Children with ADHD showed larger conditional effect in deoxyHb in the right DLPFC		
	Schecklmann et al., 2010	Smaller deactivation (oxyHb decrease) in working memory than in the control condition	n.s.	n.s.	n.s.	Activation level differed between ADHD children with and without medication in the left SFS and right DLPFC		
	Negoro et al., 2010	Task-related oxyHb increase in bilateral inferior frontal region	↓	Bilateral	inferior PFC			
	Schecklmann et al., 2011a	Significant oxyHb increase during olfactory stimulation in bilateral IFC and temporal region	↓	Bilateral	Bilateral PFC and temporal region	Significant correlation between activations in left IFC/temporal region and olfactory discrimination performance in pre-medicated children with ADHD		
	Xiao et al., 2012	NA	↓ in NoGo task ↑ in Stroop task	Right Right	Frontopolar PFC Frontopolar PFC			
	Inoue et al., 2012	Significantly larger oxyHb increase in the NoGo than in go condition	↓	Bilateral	Frontopolar PFC, VLPFC			
	Tsujimoto et al., 2013	Task-related sustained increase in oxyHb from baseline	↑	Right	Frontopolar PFC, VLPFC	Positive correlation between oxyHb in the right PFC and error rate There was significant group difference also in the middle, but not the left, channel cluster		
	Yasumura et al., 2014	Bilateral oxyHb increase in Reverse stroop task	↓	Right	Frontopolar PFC, VLPFC	Negative correlation between SNAP inattention score and oxyHB in Ch4 (Right PFC)		
	Ichikawa et al., 2014	Significantly larger increase of oxyHb in the right than in the left temporal region in response to both angry and happy expressions	↓ to angry expression	Right	Right superior temporal region	Larger variance in the timing of peak activation in the right hemisphere in boys with ADHD		
	Nagashima et al., 2014a	Significant oxyHb increase in the right MFG/IFG and right angular/supramarginal gyrus	↓	Right	IFG/MFG	The group difference between control and pre-medicated group was eliminated by the administration of MPH		
	Nagashima et al., 2014b	Significant oxyHb increase in the right MFG/IFG and right angular/supramarginal gyrus	↓ in MFG/IFG ↓ in angular/ supramarginal gyrus	Right Right	IFG /MFG Suplamarginal and angular gyrus	The group difference between control and pre-medicated ADHD was eliminated by the administration of ATX		
	Nagashima et al., 2014c	Significant oxyHb increase in the right MFG/IFG	↓ in MFG/IFG	Right	IFG/MFG	The group difference between control and pre-medicated ADHD was eliminated by the administration of ATX		
	Monden et al., 2015	Significant oxyHb increase during NoGo block in the right MFG/IFG	↓ in MFG/IFG	Right	IFG/MFG	The activation level in these regions classified ADHD children and healthy controls with high accuracy		
	Köchel et al., 2015	OxyHb increase in right temporal gyrus, but not in supramarginal gyrus in response to angry prosody	↓↑	Right Bilateral	STG Supramarginal gyrus	Hyper activation in bilateral supramarginal gyrus to anger, which the authors attribute to compensatory enhancement of attention allocation		
	Yasumura et al., 2015	Task-related OxyHb increase from baseline in the bilateral PFC	↓	Bilateral	IFG	Negative correlation between SNAP scores and oxyHb in Ch1 (right IFG) when both control and ADHD groups were considered		
	Ishii-Takahashi et al., 2015^*^	OxyHB increase during trial in the bilateral IFC	↓	Right	IFC	The hypoactivation in the left IFC approached significance		
	Araki et al., 2015	Significant task-related oxyHb increase from baseline during CPT in bilateral DLPFC	↓	Bilateral	DLPFC	The activation level in bilateral DLPFC was normalized by the administration of ATX		
Adult	Ehlis et al., 2008	Task-related increase from baseline in oxyHb in bilateral DLPFC	↓	Bilateral	DLPFC			
	Schecklmann et al., 2009	Task-related increase in oxyHb during fluency compared to control task	↓	Bilateral	DLPFC, VLPFC			
	Schecklmann et al., 2011b	Significant oxyHb increase from baseline in bilateral temporal inferior frontal and somatosensory regions	↓	Bilateral	Superior/middle temporal region	Positive correlation between oxyHb increase in the right inferior frontal ROI and sensitivity to odor sample Positive correlation between I7/WURS-k and oxyHb in bilateral temporal and somatosensory ROIs		
	Schecklmann et al., 2012	Task-related increase of oxyHb in DLPFC in working memory task. The degree of increase was significantly larger when the working memory load was larger Successful stop trials was accompanied by larger oxyHb increase in IFC than go-trials	↓ in working memory task	Bilateral	DLPFC	During SST, controls showed significant oxyHb increase in bilateral IFC in successful stop compared to go-trials, which was not the case in ADHD children		
	Ishii-Takahashi et al., 2014	NA	↓ in SST ↓ in VFT	Bilateral Right Left	Frontopolar PFC, DLPFC PMA, pre-SMA VLPFC, DLPFC			

*TDC, Typically Developed Children; TDA, Typically Developed Adults; DLPFC, Dorsolateral Prefrontal Cortex; SFS, Superior Frontal Sulcus; MPH, Methylphenidate; LPFC, Lateral Prefrontal Cortex; IFG, Inferior Frontal Gyrus; MFG, Middle Frontal Gyrus; ATX, Atomoxetine; STG, Superior Temporal Gyrus; IFC, Inferior Frontal Cortex; VLPFC, Ventral Prefrontal Cortex; SST, Stop-Signal Task; SMA, Supplementary Motor Area; PMA, Primary Motor Area; CPT, Continuous Performance Test*.

* *The results of only baseline assessment in this study are shown here*.

Most of the studies of children with ADHD show atypical patterns of oxyHb more prominently in the right hemisphere during a variety of tasks such as the reverse-Stroop task (Yasumura et al., 2014), executive attention control task (Tsujimoto et al., 2013), verbal fluency task (VFT; Schecklmann et al., 2009), Go/NoGo task (Monden et al., 2012, 2015; Xiao et al., 2012; Nagashima et al., 2014a), oddball task (Nagashima et al., 2014b,c), passive viewing of facial expression (Ichikawa et al., 2014), and emotional prosody recognition (Köchel et al., 2015). These studies have revealed hypoactivation in the right frontal lobe including the prefrontal cortex (PFC; Xiao et al., 2012; Yasumura et al., 2014), middle frontal gyrus (MFG), and inferior frontal gyrus (IFG) (Monden et al., 2012, 2015; Nagashima et al., 2014a,b,c), presumably because NIRS probes can easily be applied to the frontal region (see Table 1). These studies also found hypoactivation in the temporal (Ichikawa et al., 2014; Köchel et al., 2015) and parietal cortices (Nagashima et al., 2014b) as well.

Interestingly, a few studies found atypicality in the pattern of deoxyHb alteration in children with ADHD (Weber et al., 2005; Jourdan Moser et al., 2009). For example, Weber et al. (2005) reported larger deoxyHb increase in the left superior/middle frontal cortex in controls than children with ADHD, without group difference in oxyHb alteration. Low level of deoxyHb increase may reflect inefficient oxygen consumption due to redcued cortical activation. Thus, incorporating the findings on deoxyHb may give us more comprehensive picture about the hemispheric asymmetry in atypical neural function in people with ADHD, although these findings are sporadic at this point.

While the majority of studies that recruited children with ADHD report right-lateralized frontal hypoactivation (Monden et al., 2012, 2015; Xiao et al., 2012; Nagashima et al., 2014a,b,c; Yasumura et al., 2014), bilateral frontal hypoactivation seems more prevalent among adults with ADHD (Ehlis et al., 2008; Schecklmann et al., 2011b). The ADHD symptoms in children are reported to become less severe as they get older, which partly explains the lower prevalence rate of ADHD in adults than pediatric population (Pietrzak et al., 2006; Safren et al., 2010; Thomas et al., 2015). Considering this, the more wide-spread PFC hypoactivation in adults with ADHD raises the possibility that these patients constitute sub-group with severe form of ADHD, whose symptoms persist despite development. However, as the number of fNIRS studies of adult ADHD patients is disproportionately small, this observation requires further empirical validation.

## Lateralization in atypical neural function in ASD

ASD is an umbrella term collectively referring to heterogenous groups of individuals who share the following core symptoms: Deficits in socio-communicative ability, fixed or restricted behaviors, and repetitive patterns of behavior (APA, 2013). ASD has several sub-groups that differ in symptomatic profiles and cognitive-emotional ability such as intellectual and linguistic prowess (Lenroot and Yeung, 2013).

Since the early days of autism research, investigators have posited that the symptoms of ASD are associated with atypical left-hemisphere function, largely based on the observation that children with Kanner's autism have impaired linguistic ability (McCann, 1982). Later studies reported reduced leftward lateralization in people with ASD with (De Fossé et al., 2004) or without language delay (Floris et al., 2016). That is, people with ASD show weaker level of leftward lateralization in linguistic function than typically developed people. Furthermore, recent resting-state fMRI studies have shown weaker interhemispheric communication (Anderson et al., 2011) and an increased degree of rightward lateralization in the resting-state activity of non-language brain regions recruited during visual/tactile perception, motor-planning, and executive functioning (Cardinale et al., 2013).

To review fNIRS studies of people with ASD, we searched for relevant papers using the Scopus database. Similar criteria to that described in lateralization in atypical neural function in ADHD were adopted in selecting eligible studies. The details of these are summarized in Table 2. Most of these studies have refuted the notion of a leftward-lateralization in atypical function in ASD by showing bilateral hypoactivation in the frontal cortex including IFG/motor-related cortices (Kajiume et al., 2013), and dorsolateral PFC (DLPFC)/frontopolar PFC (Kawakubo et al., 2009; Iwanami et al., 2011; Iwanaga et al., 2013; Ishii-Takahashi et al., 2014) using tasks such as the VFT (Kuwabara et al., 2006; Kawakubo et al., 2009; Iwanami et al., 2011), mental-state reading task (Iwanaga et al., 2013), stop-signal task (SST; Ishii-Takahashi et al., 2014), and imitation task (Kajiume et al., 2013). In contrast to ADHD, no clear difference was observed between adult and pediatric population with ASD in the lateralization pattern in atypical neural function. A few of the studies showing bilateral hypoactivation report hypoactivation in wider cortical regions in the left than in the right hemisphere (Ishii-Takahashi et al., 2014). For example, Ishii-Takahashi et al. (2014) found hypoactivation during SST in the left ventrolateral PFC (VLPFC) and motor-related areas, in addition to the bilateral DLPFC/ frontopolar PFC.

**Table 2:** **The details of the main fNIRS studies on people with ASD explained in the present mini-review: only the results of group comparison with matched-controls are shown**.

	**Age**	**Model of NIRS machine**	**Number of channels**	**Task requirements**	**Measured regions**	**Dependent variables**	**Main analysis of fNIRS data**
Kuwabara et al., 2006	6 males and 4 females with PDD (*M* = 26.5 ± 7.1) 10 TDA (9 males and 1 female; *M* = 27.9 ± 4.1)	ETG-100	24	Letter fluency task	Frontal lobe	OxyHb DeoxyHb CARS Behavioral Performance	ANOVA with the factors of Group × Hemisphere × Channel
Minagawa-Kawai et al., 2009b	7 boys and 2 girls with low- or high-function ASD (*M* = 9.2± 1.8) 9 TDC (2 girls and 7 boys; *M* = 7.3 ± 1.7)	ETG-7000	8	Phonemic discrimination task Prosodic discrimination task	Temporal lobe	Laterality Quotient (LQ) of oxyHb Functional Lateralization (FL) score of oxyHb Behavioral Performance	ANOVA on FL score with the factors of Group × Task
Kawakubo et al., 2009	12 boys and 2 girls with high-functioning autism (*M* = 12.7± 3.4) 14 TDC (12 boys and 2 girls; *M* = 10.6 ± 2.8) 9 males and 4 females with high functioning autism (*M* = 26.7 ± 6.1) 13 TDA (9 males and 4 females; *M* = 25.8 ± 5.1)	NIRO-200	2	Letter fluency task	Frontal lobe	OxyHb DeoxyHb CARS Behavioral Performance	ANOVA with the factors of Group × Hemisphere for children and adults separately
Kita et al., 2011	10 boys with Asperger Syndrome or high-functioning autism (*M* = 10.2 ± 1.1) 13 TDC (13 boys; *M* = 10.9 ± 1.0)	Spectratech OEG-16	16	Self-face recognition	Frontal lobe	OxyHb Eye-movement Self-consciousness scale PARS Behavioral Performance	ANOVA on mean oxyHb values in two ROIs (L-IFG, R-IFG) with the factors of Hemisphere × Group
Iwanami et al., 2011	14 males and 6 females with Asperger syndrome (*M* = 27.2± 8.5) 18 TDA (12 males and 6 females; *M* = 31.1 ± 4.7)	ETG-4000	52	Letter and category fluency task	Frontal lobe, Temporal lobe	OxyHb AQ Behavioral Performance	ANOVA on mean oxyHb values in each task with the factors of Group × ROI (left/right temporal, frontal)
Tamura et al., 2012	16 boys and 4 girls with Asperger Syndrome or PDD (*M* = 10.2 ± 3.4; 6 autistic disorder, 9 Asperger, 5 PDD) 20 TDC (16 boys and 4 girls; *M* = 9.5 ± 2.5)	NIRO-200	2	Anatomical Imitation (AI) task Mirror-Image Imitation (MI) task	Frontal lobe	Differential value of oxyHb and deoxyHb between AI and MI (AI-MI)	ANOVA on differential oxyHb with the factors of Group × Hemisphere
Xiao et al., 2012	19 boys with high-functioning autism (*M* = 10.11± 2.08) 16 TDC (16 boys; *M* = 9.69 ± 1.74)	JH-NIRS-BR-05	16	Go/NoGo task Stroop task	Frontal lobe	OxyHb Behavioral Performance	Group comparison of mean oxyHb in each hemisphere by *t*-tests
Funabiki et al., 2012	10 males and 1 female with Asperger Syndrome or PDD without language delay (*M* = 16.8± 6.1) 12 TDC (10 boys and 2 girls; *M* = 14.2 ± 3.8)	OMM-3000	32	Intentional listening or ignoring tones or stories	Frontal lobe, Temporal lobe	OxyHb DeoxyHb Behavioral Performance	ANOVA on mean oxyHb values in PFC and temporal region with the factors of Group x Hemisphere × Attentional State
Narita et al., 2012	3 males and 8 females with ASD (*M* = 29.5, range = 14–46) Typically developed people (6 males and 16 females; *M* = 25.2, range = 19–51)	NIRO-200	2	Visuo-spatial working memory task	Frontal lobe, Temporal lobe	OxyHb Behavioral performance	Comparison of conditional differences in each group
Iwanaga et al., 2013	14 boys and 2 girls with ASD (*M* = 11.5 ± 1.8) 16TDC (12 boys and 4 girls; *M* = 11.4 ± 1.8)	ETG-4000	22	Mental State (MS) task Object Characteristics (OC) task	Frontal lobe	OxyHb Behavioral performance	ANOVA on mean oxyHb values in two ROIs (left/right MPFC) with the factors of Group × Hemisphere × Task
Kajiume et al., 2013	6 boys with PDD (*M* = 10.9 ± 1.6; 3 PDD-NOS, 3 Asperger Syndrome) 6 TDC (6 boys; *M* = 10.9 ± 1.6)	ETG-100	24	Imitation task Observation task	Frontal lobe, Temporal lobe	OxyHb DeoxyHb Social skill test	Channel-wise Analysis using ANOVA with the factors of Group × Task
Yasumura et al., 2014	7 boys and 4 girls with ASD (*M* = 10.51± 2.3) 15 TDC (6 boys and 9 girls; M = 28.8 ± 5.5)	ETG-100	24	Stroop task Reverse stroop task	Frontal lobe	OxyHb SNAP questionnaire Behavioral performance	ANOVA on mean oxyHb in each hemisphere with the factors of Hemisphere × Group
Ishii-Takahashi et al., 2014	8 males and 13 females with ASD (*M* = 30.8 ± 7.2; 5 Asperger Syndrome and 16 PDD-NOS) 21 TDA (13 males and 8 females; *M* = 28.8 ± 5.5)	ETG-4000	52	VFT SST	Frontal lobe, Temporal lobe	OxyHb Behavioral performance	Channel-wise group comparison of oxyHb Classification of groups by linear discriminant analysis using oxyHb
Jung et al., 2016	8 people with ASD (*M* = 15.6 ± 9.55) 12 typically developed males (*M* = 14.5 ± 10.8)	TechEn CW6 fNRIS system	14	1-back task using pictures of Human and robot face	Temporal lobe	OxyHb GARS-2 score	ANOVA with the factors of Group × Hemisphere for human and robot face
	**Typical activation pattern**	**Patients compared to controls**	**Hemisphere**	**Regions with group difference**	**Other Findings**		
Kuwabara et al., 2006	Significant task-related increase of OxyHb in bilateral PFC	↓	Bilateral	PFC	oxyHb in the right PFC correlated negatively with CARS verbal communication score		
Minagawa-Kawai et al., 2009b	Larger FL score in phonemic than in prosody discrimination task	n.s.	n.s.	n.s.	Significantly smaller FL score in children with ASD than in controls in phonemic discrimination task		
Kawakubo et al., 2009	OxyHb increase during letter fluency task	↓ in adults	Bilateral	Ventral PFC			
Kita et al., 2011	Slight oxyHb increase in typically-developed children, which was significantly smaller than in typically developed adults	n.s.	n.s.	n.s.	OxyHb in R-IFG correlated positively with the level of public self-consciousness and negatively with ASD severity		
Iwanami et al., 2011	OxyHb increase during both tasks. The amplitude is larger in letter than category fluency task	↓ in letter fluency task	Bilateral	Frontopolar PFC, DLPFC, VLPFC, and Superior Temporal region			
Tamura et al., 2012	Larger differential value of oxyHb in the left than in the right hemisphere	n.s.	n.s.	n.s.	No hemispheric asymmetry was observed in ASD		
Xiao et al., 2012	NA	↓ in GoNoGo task	Right	Frontopolar PFC			
Funabiki et al., 2012	Larger oxyHb increase in the temporal region when the participants listened to auditory stimuli intentionally	n.s.	n.s.	n.s.	Significant interaction between Hemisphere and Attentional state in story listening in PFC only in ASD group		
Narita et al., 2012	Larger oxyHb level during Working Memory (WM) compared to Non-Working Memory (NWM) condition. The overall level of oxyHb level increased as the task load increased	n.s.	n.s.	n.s.	ASD children failed to show clear WM>NWM pattern in oxyHb		
Iwanaga et al., 2013	OxyHb increase in bilateral MPFC	↓ in MS task	Bilateral	MPFC			
Kajiume et al., 2013	Task-related oxyHb increase	↓ in action observation	Bilateral (mostly in the right)	IFG/PMC			
Yasumura et al., 2014	Bilateral oxyHb increase in Reverse stroop task	n.s.	n.s.	n.s.			
Ishii-Takahashi et al., 2014	NA	↓ in SST ↓ in VFT	Bilateral Left Left	DLPFC VLPFC, PMA, SMA VLPFC, DLPFC			
Jung et al., 2016	Significantly larger increase of oxyHb in the right than left temporal region to human faces. No hemispheric asymmetry was observed to robot faces	n.s.	n.s.	n.s.	ASD children did not show hemispheric asymmetry in oxyHb level to human faces		

*TDC, Typically Developed Children; TDA, Typically Developed Adults; DLPFC, Dorsolateral Prefrontal Cortex; IFG, Inferior Frontal Gyrus; VLPFC, Ventral Prefrontal Cortex; MTG, Middle Temporal Gyrus; PMA, Primary Motor Area; SMA, Supplementary Motor Area; MPFC, Medial Prefrontal Cortex*.

Several studies report reduced lateralization in neural function in people with ASD. For example, Minagawa-Kawai et al. (2009b) reported weaker leftward-lateralization of oxyHb increases in Wernicke areas when children with ASD engaged in a phonemic discrimination task, although they did not report the results of direct group-comparisons of task-related oxyHb changes. Likewise, Jung et al. (2016) reported that people with ASD failed to show rightward-lateralization of oxyHb increases in the posterior temporal region in response to human faces as was observed in the matched control group.

One potential reason for such inconsistency among previous studies is the symptomatic heterogeneity of ASD. ASD has several sub-groups that differ in symptomatic profiles and cognitive-emotional ability (Lenroot and Yeung, 2013). The left-hemisphere theory of ASD was originally proposed for individuals with Kanner's autism with language delay (McCann, 1982). However, most fNIRS studies have recruited people with high-functioning autism or Asperger Syndrome (Kawakubo et al., 2009; Iwanami et al., 2011; Kita et al., 2011; Xiao et al., 2012; Iwanaga et al., 2013; Yasumura et al., 2014), possibly due to the task requirements, with rare exceptions (Minagawa-Kawai et al., 2009b). Considering this, it is possible that evidence supporting a clearer pattern of lateralization can be obtained for specific sub-groups.

## General discussion

Existing fNIRS studies generally support the notion of the right-lateralization in atypical function in children with ADHD. The use of fNIRS for the clinical examination of children is promising, especially because the exclusion rate for fNIRS measurement is reported to be much lower than that for fMRI (Nagashima et al., 2014b). This potential has been gainfully exploited by Monden et al. (2012, 2015), who assessed the efficacy of a pharmacological intervention in children with ADHD using oxyHb increases in the right PFC as an indicator (see also, Nagashima et al., 2014a,b,c). Future research should estimate the sensitivity/specificity of right PFC activation as a biomarker of ADHD (Monden et al., 2015) and investigate whether the rightward-lateralization in atypical activation is uniquely linked to ADHD. We did not find a clear pattern of leftward-lateralization in atypical function for people with ASD. As noted above, this is partly because of the heterogeneity of people with ASD.

There remain several unresolved issues important for the further development of research on the lateralization in atypical neural function. The first is the establishment of a standard analytic method. As summarized in the tables, the analytic approach for multi-channel fNIRS data can be classified into two groups. One is the region-of-interest (ROI) approach, in which neighboring channels are grouped into single ROI and the averaged levels of oxyHb in channels within ROI are analyzed as the main indicators of neural activation. In this approach, corresponding channels in the left and right hemisphere are usually integrated into left/right ROI. The other approach is channel-wise analysis, in which a primary statistical test is conducted for each channel. It is unclear at this point which of these two approaches is more advantageous for detecting lateralized patterns in atypical activation. Channel-wise analysis is more sensitive to highly localized group-differences than the ROI approach, and thus might be more suitable for detecting signs of lateralization in atypical function. The main problem of the channel-wise approach is how to set the significance threshold. Apparently, a large number of statistical tests leads to inflation of the false-positive rate, while a conventional method for adjusting the threshold, e.g., Bonferroni's procedure, is sometimes too stringent.

The second issue also relates to the analytic procedure. There are several problems in the group comparisons of fNIRS results. First, due to morphological variations in cortical structure, the location and depth of the cortical region through which the infrared light passes might differ between people with and without developmental disorders. Second, it is often noted that children with ADHD/ASD show larger bodily and facial movements than matched controls during experimental tasks, which might introduce group-differences in the level of artifacts and consequently influence the results. Especially problematic is the artifact of skin blood perfusion accompanying facial muscle contractions (Takahashi et al., 2011; Seiyama et al., 2016). To overcome these problems inherent in group-comparisons of fNIRS signals is surely an important agenda for future research.

The third point is the scarcity of fNIRS studies on resting state activation (Medvedev, 2014). Of particular relevance, one of the strongest pieces of evidence for the left-hemisphere theory of ASD comes from a resting state activation study (Cardinale et al., 2013). Thus, more research should focus on the patterns of lateralization of oxy-/deoxy-Hb alteration in the resting state. One of the most popular approaches to characterizing resting state activity is the analysis of inter-region functional connectivity. Several fNIRS studies have tried to characterize neural function in developmental disorders (Kikuchi et al., 2013; Zhu et al., 2015; Li and Yu, 2016; Li et al., 2016), and interestingly, several of them found lateralized patterns of atypical connectivity in the patient group (Zhu et al., 2015). We did not review fNIRS studies of functional connectivity in people with ADHD or ASD, as the analysis procedures vary greatly between studies and the number of eligible studies is too small to draw any coherent conclusions. However, considering the rapid development of this field of research, our knowledge of the lateralization in atypical function is further enriched by this novel approach.

The fourth is the potential confound of medication. The number of studies recruiting only drug-naïve patients is relatively few and participants in the patient group are taking various kinds of medications in majority of the studies. Fuethremore, several fNIRS studies reviewed above have shown that short-term administration of drugs such as methylphenidate changed the pattern of cortical activation in children with ADHD (Nagashima et al., 2014a,b,c; Monden et al., 2015). On the basis of these, more studies recruiting only non-medicated patients are needed to clarify the precise nature of atypicality in neural function.

## Conclusion

In this mini-review, we gave a brief overview of the findings of fNIRS studies about lateralization in atypical neural function in people with ADHD and ASD. The existing studies generally support rightward-lateralization in atypical function in children with ADHD. At the same time, we did not find clear pattern of the leftward-lateralization in atypical function for people with ASD. Nevertheless, lateralization in atypical neural function might have been obscured by factors such as sample heterogeneity and particular method of analysis.

## Author contributions

HD conceived this study. HD and KS wrote the manuscript.

### Conflict of interest statement

The authors declare that the research was conducted in the absence of any commercial or financial relationships that could be construed as a potential conflict of interest.
